# Sustainable Sources of Raw Materials for Additive Manufacturing of Bone‐Substituting Biomaterials

**DOI:** 10.1002/adhm.202301837

**Published:** 2023-08-10

**Authors:** Niko E. Putra, Jie Zhou, Amir A. Zadpoor

**Affiliations:** ^1^ Department of Biomechanical Engineering Faculty of Mechanical Maritime and Materials Engineering Delft University of Technology Mekelweg 2 Delft 2628 CD The Netherlands

**Keywords:** 3D printing of implants, additive manufacturing, orthopedic biomaterials, sustainability

## Abstract

The need for sustainable development has never been more urgent, as the world continues to struggle with environmental challenges, such as climate change, pollution, and dwindling natural resources. The use of renewable and recycled waste materials as a source of raw materials for biomaterials and tissue engineering is a promising avenue for sustainable development. Although tissue engineering has rapidly developed, the challenges associated with fulfilling the increasing demand for bone substitutes and implants remain unresolved, particularly as the global population ages. This review provides an overview of waste materials, such as eggshells, seashells, fish residues, and agricultural biomass, that can be transformed into biomaterials for bone tissue engineering. While the development of recycled metals is in its early stages, the use of probiotics and renewable polymers to improve the biofunctionalities of bone implants is highlighted. Despite the advances of additive manufacturing (AM), studies on AM waste‐derived bone‐substitutes are limited. It is foreseeable that AM technologies can provide a more sustainable alternative to manufacturing biomaterials and implants. The preliminary results of eggshell and seashell‐derived calcium phosphate and rice husk ash‐derived silica can likely pave the way for more advanced applications of AM waste‐derived biomaterials for sustainably addressing several unmet clinical applications.

## Graphical abstract

Recycling waste for biomaterials offers an avenue for the sustainable development of medical devices. Waste, such as eggshells, marine residues, and agricultural biomass can be transformed into bone substitutes. Probiotics and renewable polymers can enhance the biofunctionality of the biomaterials, while additive manufacturing (AM) enables sustainable production. AM waste‐derived biomaterials are expected to sustainably address current clinical challenges.

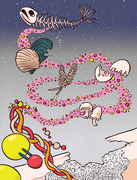

## 1 Introduction

The rapid growth of human population has intensified the negative impact of economic activities on the environment, leading to an increase in environmental issues, such as loss of biodiversity, environmental pollution of air, water, and soil, as well as the depletion of natural resources. These issues are often the result of mass‐scale agricultural and industrial activities, including those in the healthcare sector, which generate billions of tons (unregulated) waste that is harmful to the environment.^[^ ^1^ ^]^ This pressing environmental challenge calls for rigorous research into sustainable sources of raw materials that can, e.g., be obtained by repurposing existing waste into functional medical devices, such as bone substitutes and orthopedic implants.

Bone has a high self‐healing capability. However, a large‐scale damage beyond a species‐dependent critical size requires an implant to guide its regeneration process. Every year, a few million bone‐grafting procedures are performed.^[^ ^2^ ^]^ Despite it being the gold standard for bone substitution, there are multiple challenges associated with the use of autologous bone grafts. For example, the available bony stock is limited. Moreover, patients face morbidity at the bone graft harvest site.^[^ ^3^ ^]^ Finally, the additional surgery required for bone harvesting leads to major complication rates in 8.6–17.9% patients undergoing such surgeries.^[^ ^4^, ^5^ ^]^ Not surprisingly, the demand for synthetic bone implants is high and is expected to further increase as the world's population ages. For example, the number of patients requiring bone implants due to osteoporotic fractures is expected to double by 2040.^[^ ^6^ ^]^ These conditions create a global market for synthetic bone implants with an annual turnover exceeding 100 billion US$.^[^ ^7^ ^]^

As far as permanent bone implants, such as those used for total joint replacements, are concerned, the primary research focus is placed on increasing the implant longevity through enhanced osseointegration, improved bony ingrowth, infection prevention, and effective treatment of implant‐associated infections.^[^ ^8^, ^9^ ^]^ These efforts aim to ensure that the permanent bone implant functions for the entirety of the patient's life, fulfilling one of the sustainability pillars for maximizing a material's lifetime.^[^ ^10^ ^]^ In other cases, such as transverse bone fractures, biodegradable implants may be used instead of the permanent ones to provide mechanical support during the initial healing stage of bone defects.^[^ ^11^ ^]^ As bone tissue heals and regains its original strength and function, the implant biodegrades in the body. While this type of implant is not sustainable with regard to the material's lifetime, it can be seen as a sustainable choice for the patient and for the environment, because it eliminates the need for a second surgery of the patient to remove the implant and its associated potential surgical complications.^[^ ^12^ ^]^ This ultimately decreases not only healthcare costs but also the considerable hospital waste associated with surgeries and post‐surgery hospital care.

On average, a single orthopedic surgery generates 6.2 kg of waste,^[^ ^13^ ^]^ while an arthroplasty procedure produces a greater amount of waste ranging between 13.6 and 15.1 kg per case.^[^ ^14^ ^]^ With around 7 million orthopedic procedures performed annually in the US alone,^[^ ^13^ ^]^ the amount of waste generated is ≈43 000–106 000 tons per year. In the UK, the National Health Service (NHS) generates about 25 megatons of CO_2_ emissions and produces more than 500 000 tons of waste every year.^[^ ^14^ ^]^

Unfortunately, both permanent and biodegradable implants often require the use of nonrenewable and unsustainable materials for their fabrication, such as fossil‐derived polymers or materials obtained through mining for metallic and limestone minerals. In the case of mined materials, the processes of mining, purifying, alloying, heat treatment, and prefabrication (e.g., creating metal powder from ingots) are all associated with significant energy expenditure and, thus, a major carbon footprint.^[^ ^15^, ^16^ ^]^

The bone implants used in clinical settings are typically made of metals (e.g., titanium (Ti)‐based materials), synthetic polymers, hydroxyapatite (HA), other calcium phosphate‐based bioceramics, and bioactive glass.^[^ ^17^, ^18^, ^19^ ^]^ Now, new categories of biomaterials are emerging for clinical application, including biodegradable bone screws made of magnesium (Mg)‐based materials^[^ ^20^, ^21^ ^]^ and some other types of such medical devices that have been approved for clinical studies in various countries.^[^ ^22^ ^]^ Meanwhile, the research on (3D printed) iron (Fe) and zinc (Zn)‐based (porous) bone implants is still at the preclinical stages.^[^ ^23^, ^24^, ^25^ ^]^ As previously mentioned, such biomaterials address some sustainability issues. However, the research into sustainable biomaterials for orthopedic applications has been highly limited so far. One way to consider sustainability in the development of biomaterials is to use natural polymers. These materials are one of the most sustainable types of biomaterials due to their abundancy, self‐renewability, and the fact that their waste can be recycled for reuse.^[^ ^10^ ^]^ Research on natural polymers and their composites for bone tissue engineering has highlighted the use of polysaccharides (e.g., cellulose, bacterial cellulose,^[^ ^26^ ^]^ alginate,^[^ ^27^ ^]^ and crustacean chitin^[^ ^28^ ^]^), natural fibers from bamboo or coir,^[^ ^29^ ^]^ and proteins, (e.g., marine collagen,^[^ ^30^ ^]^ silk fibroin,^[^ ^31^ ^]^ and recycled keratin^[^ ^32^ ^]^).

Some other categories of orthopedic biomaterials, such as calcium phosphate‐based bone implants, lend themselves to additional avenues of design and manufacturing for sustainability. For example, recycling industrial waste, such as eggshells,^[^ ^33^, ^34^ ^]^ bi‐valve shells,^[^ ^35^ ^]^ and fish bone^[^ ^36^ ^]^ may be considered as sustainable source raw materials for the development of calcium phosphate‐based biomaterials. The main component of bioactive glass (SiO_2_‐P_2_O_5_ Na_2_O‐CaO) and silica (SiO_2_) can be extracted from agricultural waste.^[^ ^37^ ^]^ Meanwhile, recycled metals have been used in various industries, such as building and construction, industrial machinery, automotive, and shipbuilding, but not yet for the fabrication of implantable medical devices, including bone implants.

Transforming various types of wastes into biomaterials has been explored with a focus on extraction and processing steps for added functionalities.^[^ ^38^ ^]^ The current recycling step typically involves: i) converting the waste (e.g., eggshells, crustacean shells, seashells, or marine residues) and polymer biomass (e.g., rice husks or wheat straw) into the powder, pellet, paste, or liquid forms, ii) subjecting the waste to chemical or heat treatments to obtain materials with the desired phase compositions (e.g., turning eggshells into calcium phosphate powder, chitin into chitosan, marine residue into collagen, or pyrolyzing biomass into biochar containing SiO_2_ bioactive materials), and then iii) using the treated materials as the starting material for the fabrication of bone implants, often using powder metallurgy techniques.

There has been limited research on the use of additive manufacturing (AM, also known as 3D printing) for the fabrication of waste‐derived bone implants. AM is inherently sustainable due to its additive nature and has the potential to provide a closed‐loop supply chain for the on‐demand manufacturing of medical devices, such as patient‐specific orthopedic implants.^[^ ^39^ ^]^ While AM is not a waste‐free manufacturing technique, it generates less waste as compared to many other traditional processes in general and subtractive manufacturing processes in particular.^[^ ^40^ ^]^ AM places the exact amount of material exactly in the product where it is needed. AM also offers precise, controlled fabrication of complex porous designs with bespoke geometries and topologies, making it suitable for the production of functional bone implants.^[^ ^41^ ^]^

This review presents the recent advances in transforming renewable and waste materials into biomaterials for bone tissue engineering. We also examine the use of AM to process waste‐material‐sourced raw materials into bone implants and provide several suggestions for using multimaterial AM to improve the functionality of waste‐derived and renewable biomaterial for bone implant applications.

## 2 Sustainable Resources for Additive Manufacturing of Bone Implant Materials

Human tissue and organ repair can be aided by various biomaterials, such as bioactive ceramics, bioactive glasses, natural polymers, metals, and their composites.^[^ ^42^ ^]^ Sustainable resourcing of components for the fabrication of regenerative biomaterials, including bone implants, is important to improve the circularity in the use of such materials and the conservation of natural mineral resources that take hundreds of thousands to millions of years to form.

### 2.1 Recycled Calcium Phosphate Bioceramics from Poultry and Seafood Wastes

Calcium phosphate bioceramics are the most commonly used bone‐substituting materials, with nanocrystalline HA and β‐tricalcium phosphate (TCP) bioceramics being widely studied^[^ ^43^, ^44^ ^]^ and used in clinical settings.^[^ ^17^, ^18^ ^]^ These materials can be derived from the calcium‐rich waste generated by the poultry and seafood industries. In 2018, it was estimated that there were around 8.6 million tons of eggshell waste produced,^[^ ^45^ ^]^ ranking it as the 15th most polluting waste product.^[^ ^45^ ^]^ The seafood industry generates 6–8 million tons of shell waste per year,^[^ ^46^ ^]^ while only 9% of the global seashell waste is recycled as additive in fertilizers and animal food.^[^ ^47^ ^]^

#### 2.1.1 Calcium Carbonate from Eggshells and Seashells

**Figure 1:**
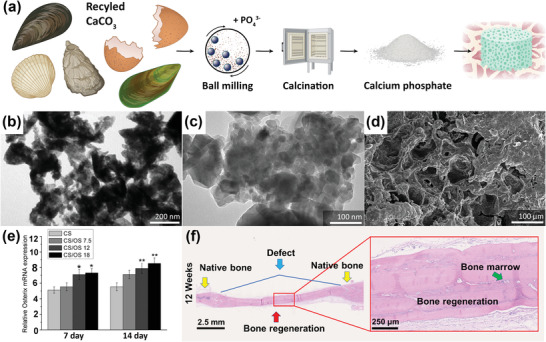
Eggshell and seashells for recycled Ca‐based bioceramics. a) A schematic illustration of sustainably sourced CaCO_3_ and the material processing steps to obtain calcium phosphate powder and porous 3D scaffold. Created with BioRender.com. b) Eggshell‐derived HA particles synthesized by solid‐state sintering. Reproduced with permission.^[^ ^53^ ^]^ Copyright 2016, Wiley‐VCH GmbH. c) Aragonite NPs from bivalve mollusk shells. Reproduced under the term of CC‐BY license.^[^ ^80^ ^]^ Copyright 2017, The Authors. Published by Elsevier. d) Porous aragonite NPs composites containing gelatin, dextran, and dextrin. Reproduced under the term of CC‐BY license.^[^ ^80^ ^]^ Copyright 2017, The Authors. Published by Elsevier. e) The elevated Osterix gene expression of bone cells exposed to calcium sulfate supplemented with oyster shells. Reproduced with permission.^[^ ^77^ ^]^ Copyright 2014, American Chemical Society. f) The hematoxylin and eosin (H&E) staining on eggshell particle reinforced scaffolds indicating bone regeneration on critical‐sized cranial rat bone defects at 12 weeks. Reproduced with permission.^[^ ^57^ ^]^ Copyright 2021, American Chemical Society.

Eggshells are a readily renewable source of calcium that can be found in many households. They contain ≈96% CaCO_3_, 1% MgCO_3_, 1% Ca_3_(PO_4_)_2_, and organic matter.^[^ ^48^ ^]^ The microstructure of eggshells contains polycrystals of calcite in a columnar form with a diameter of 70 to 80 µm and a length of about 330 µm.^[^ ^49^ ^]^ Eggshells can be crushed, ground, and sieved into powder particles of various sizes. When heated to 900 °C, eggshell powder transforms into CaO. This CaO powder can then be reacted with phosphate sources, such as H_3_PO_4_,^[^ ^50^, ^51^ ^]^ NaHPO_4_,^[^ ^52^ ^]^ NH_4_H_2_PO_4_,^[^ ^53^, ^54^ ^]^ CaHPO_4_, or Ca_2_P_2_O_7_^[^ ^55^ ^]^ using chemical precipitation, solid‐state sintering, or hydrothermal treatment (**Figure** **1**a) to create calcium phosphate powders, like HA or β‐TCP (Figure 1b). Both raw eggshell and the eggshell‐derived calcium phosphate powders have been used as bone‐substituting materials. They are eggshell‐gelatin methacryloyl (GelMA),^[^ ^56^, ^57^ ^]^ nanoHA‐collagen,^[^ ^58^ ^]^ and nanoHA‐cellulose composites,^[^ ^59^ ^]^ brushite bone cements,^[^ ^60^ ^]^ β‐TCP foams,^[^ ^61^ ^]^ nanofibrous polycaprolactone (PCL) and polyvinyl alcohol‐TCP composites,^[^ ^62^ ^]^ and bioglass ceramics containing calcium phosphate.^[^ ^63^ ^]^

Seashells are primarily composed of CaCO_3_, similar to eggshells. However, the microstructure of seashells exhibits great complexity due to the diverse shell types found across various mollusk species. Typically, seashells comprise multiple calcified layers, consisting of different forms of CaCO_3_, such as calcite or aragonite. These CaCO_3_ crystals can be arranged in intricate nanoscale structures, including nacreous, prismatic, foliated, and cross‐lamellar, which are almost impossible to replicate synthetically.^[^ ^64^ ^]^ Moreover, this complex structure arrangement makes the minerals up to 3000 times stronger than single crystals.^[^ ^65^ ^]^ Using the same processing methods as the ones used for eggshells, seashells, which contain up to 95% CaCO_3_ (Figure 1c), can be transformed into various types of calcium phosphate bioceramics.^[^ ^66^, ^67^, ^68^, ^69^, ^70^, ^71^, ^72^, ^73^, ^74^, ^75^ ^]^ Seashell powders have been used to create porous biphasic calcium phosphate scaffolds,^[^ ^76^ ^]^ CaSO_4_‐shells composites,^[^ ^77^ ^]^ HA/β‐TCP‐chitosan composites,^[^ ^78^ ^]^ HA microspheres,^[^ ^79^ ^]^ porous aragonite (Figure 1d),^[^ ^80^ ^]^ and HA scaffolds.^[^ ^81^ ^]^ These recycled Ca‐rich biomaterials (e.g., CaSO_4_ supplemented by oyster shell particles) has been shown to improve the osteogenic gene expression (Figure 1e)^[^ ^77^ ^]^ while a hydrogel containing eggshell particles has been found to favor de novo bone generation in vivo (Figure 1f).^[^ ^57^ ^]^

#### 2.1.2 Calcium Phosphate and Collagen from Fish Residues

**Figure 2:**
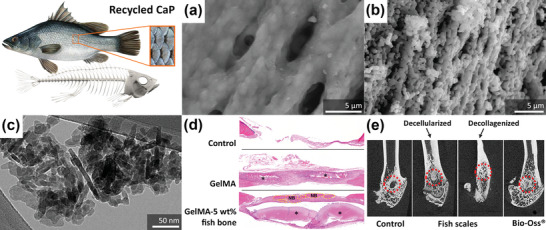
Fish residues for recycled bone‐substituting materials. The morphologies of a) pristine fish bone and b) fish bone‐derived HA. Reproduced with permission.^[^ ^91^ ^]^ Copyright 2015, Elsevier. c) Fish scale‐derived nanoHA. Reproduced with permission.^[^ ^98^ ^]^ Copyright 2019, Elsevier. d) The H&E staining showed the new bone formation (indicated by NB) on the tissue implanted with GelMA composites containing nanofish bone at 4 weeks in vivo. Reproduced with permission.^[^ ^93^ ^]^ Copyright 2020, American Chemical Society. e) Micro‐computed tomography (CT) scans of in vivo bone regeneration in a rat femoral defect model, showing the effects of decellularized and decollagenized fish scales on new bone formation at the bone defect site (indicated by the dashed red circle). Adapted with permission.^[^ ^95^ ^]^ Copyright 2023, Elsevier.

In addition to the use of eggshells and seashells, fish residues can be an alternative material source for bone repair. Fish bones, which are made of 60% calcium orthophosphate,^[^ ^36^ ^]^ can be refined through heat treatment to achieve a desired phase composition, such as HA or TCP (**Figure** **2**a,b).^[^ ^82^, ^83^, ^84^, ^85^, ^86^, ^87^, ^88^, ^89^, ^90^ ^]^ The pristine or treated fish bone powders have been used to fabricate porous HA^[^ ^91^ ^]^ and porous biphasic calcium phosphate scaffolds.^[^ ^92^ ^]^ They can also be incorporated into hydrogels, such as GelMA,^[^ ^93^ ^]^ and alloys, such as Mg‐Zn,^[^ ^94^ ^]^ to form composites. The addition of 5 wt% fish bone to GelMA has been reported to contribute de novo bone formation in vivo (Figure 2d).^[^ ^93^ ^]^

Fish scales contain up to 45% of organic components (e.g., collagen, lecithin, sclerotin, and vitamins) and up to 46% of inorganic components (e.g., calcium‐deficient HA and calcium phosphate), as well as trace elements. This composition is similar to that of the human bone tissue.^[^ ^95^ ^]^ Fish scale can be converted into HA powder (Figure 2c)^[^ ^96^, ^97^, ^98^ ^]^ and into porous HA scaffolds following the powder metallurgy route.^[^ ^99^, ^100^ ^]^ Fish scales have been found to significantly upregulate anti‐inflammatory and pro‐healing cytokines in vivo, thereby promoting bone healing (Figure 2e). In addition to the inorganic components, the collagens in fish scales are arranged in a Bouligand microstructure, resembling a revolving staircase‐like structure. This microarchitecture provides them with a high toughness, making them suitable for application in transitional tissue repair. For example, the in situ mineralized calcium silicate on fish scales has been shown to restore the tendon–bone interface in rat and rabbit rotator cuff tear models.^[^ ^101^ ^]^

When comparing eggshells, seashells, and fish residue as potential sources of calcium, eggshells have the advantage of being readily available and containing CaCO_3_ of a more uniform composition and microstructure.^[^ ^49^ ^]^ The microstructural diversity of seashells allows for customization and tailoring of the resulting properties of calcium‐based bioceramics. While these three types of waste materials, i.e., eggshells, seashells, and fish residues, are all rich in calcium suitable for bone tissue repair, fish bones possess the additional advantage of combining the organic matrix of collagen with inorganic calcium phosphate minerals,^[^ ^87^ ^]^ thereby offering a composition that could more closely mimic the human bone tissue. Furthermore, the Bouligand microstructure of fish scale provides a high toughness property and is promising for further development in the field of hard–soft interface biomaterials.^[^ ^101^ ^]^

### 2.2 Recycled Silica and Bioactive Compounds from Agricultural Wastes

SiO_2_‐based biomaterials are an attractive option for bone implants because of their excellent bioactivity and tunable biodegradability, which supports bone regeneration and infection prevention. Calcium silicate‐based materials are a naturally occurring group of limestones that can be produced by reacting CaO and SiO_2_ at various ratios, such as wollastonite (CaO‐SiO_2_) or calcium orthosilicate (2CaO‐SiO_2_). Bioactive ceramics made from calcium silicate have been shown to enhance bone regeneration in vivo,^[^ ^102^, ^103^ ^]^ outperforming clinically used calcium phosphate bioceramics.^[^ ^104^ ^]^ Additionally, adding MgO into the CaO/SiO_2_ binary system can create monticellite (CaO‐MgO‐SiO_2_), diopside (CaO‐MgO‐2SiO_2_), akermanite (2CaO‐MgO‐2SiO_2_), merwinite (3CaO‐MgO‐2SiO_2_), and bredigite (7CaO‐MgO‐4SiO_2_), which has been shown to further improve the biofunctionalities of the calcium silicate bioceramics. MgO is more stable than CaO, which delays the solubility of the bioceramics and extends the initial mechanical integrity of the material while providing osteogenic properties.^[^ ^105^, ^106^ ^]^

#### 2.2.1 Silica from Rice Husk Ash

**Figure 3:**
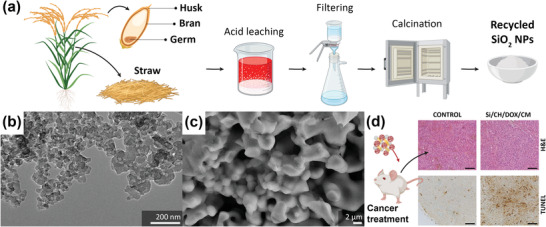
Agricultural residue for recycled SiO_2_‐rich bioceramics. a) A schematic illustration of rice plant and its recycling processes to obtain SiO_2_ NPs. Created with BioRender.com. b) Biogenic SiO_2_ NPs from rice husk calcined at 500 °C. Reproduced with permission.^[^ ^111^ ^]^ Copyright 2014, Elsevier. c) The morphology of wollastonite scaffold synthesized from rice husk ash and limestone. Reproduced with permission.^[^ ^117^ ^]^ Copyright 2018, Elsevier. d) The H&E and terminal deoxynucleotidyl transferase‐mediated dUTP nick‐end labeling staining of mice tumor tissue treated with SiO_2_ nanocarriers coated with chitosan (CH), loaded with doxorubicin (DOX) and then functionalized with cell membrane (CM). Scale bar = 100 µm. Reproduced with permission.^[^ ^124^ ^]^ Copyright 2023, Elsevier.

Grains like rice and wheat are among the world's most popular food crops. Agricultural activities, which aim at sustaining the global food supply, generate large volumes of agro‐waste that are often burnt in open air and contribute to pollution. Interestingly, grain plants have the ability to accumulate amorphous SiO_2_ and other inorganic bioactive compounds in their tissue, offering the potential to recycle their residues into bioactive materials (**Figure** **3**).^[^ ^107^, ^108^ ^]^

Agricultural residues, such as rice husk, contain a high level of SiO_2_ and low concentrations of other compounds, such as CaO, Fe_2_O_3_, MgO, and K_2_O.^[^ ^109^ ^]^ Rice husk biochar with 80–97% SiO_2_^[^ ^109^ ^]^ has received particular attention for SiO_2_ extraction.^[^ ^110^ ^]^ The process of obtaining high purity SiO_2_ particles involves a pyrolysis process and then acid leaching to dissolve undesired metallic elements, followed by calcination at a temperature between 500 and 700 °C (Figure 3a,b).^[^ ^111^ ^]^ Rice husk biochar, typically containing 48.65–54.59% carbon^[^ ^112^ ^]^ and other elements (i.e., hydrogen (H), oxygen (O), nitrogen (N), phosphorus (P), potassium (K), and silicon (Si)), has been processed into various types of bioactive ceramics for bone tissue engineering, such as bioglass,^[^ ^113^, ^114^ ^]^ wollastonite (Figure 3c),^[^ ^115^, ^116^, ^117^, ^118^, ^119^, ^120^ ^]^ forsterite (Mg_2_SiO_4_),^[^ ^120^ ^]^ and diopside.^[^ ^120^, ^121^ ^]^

Rice husk biochar can be mixed with other ingredients (e.g., NaOH as a sodium source or NH_4_H_2_PO_4_ as a phosphate source, or CaO from eggshell powder) at different concentrations using the chemical precipitation method, sol–gel technique, or solid‐state sintering to obtain the desired bioceramics. For example, bioactive glass can be designed to have 50% SiO_2_, 25% Na_2_O, and 25% CaO or 60% SiO_2_, 34% CaO, and 6% P_2_O_5_.^[^ ^113^, ^114^ ^]^ A simple mixture of rice husk‐derived SiO_2_ and CaO at equimolar ratio results in wollastonite.^[^ ^115^ ^]^ Several studies have also biofunctionalized wollastonite with silver (Ag) or copper (Cu) doping for additional antibacterial properties.^[^ ^116^, ^117^, ^118^ ^]^ Moreover, blending MgO with rice husk‐derived SiO_2_ at a ratio of 2:1 results in forsterite, while mixing eggshell‐derived CaO, MgO, and rice husk‐derived SiO_2_ at a ratio of 1:1:2 results in diopside.^[^ ^120^ ^]^

All of the rice husk‐derived SiO_2_ bioactive ceramics have shown in vitro biodegradability and the ability to induce apatite formation in simulated body fluid.^[^ ^114^, ^115^, ^116^, ^117^, ^118^, ^119^, ^120^, ^121^ ^]^ While chemically synthesized mesoporous SiO_2_ nanoparticles (NPs) have been widely evaluated in vivo,^[^ ^122^ ^]^ the in vivo studies on agriculturally derived SiO_2_ NPs are limited. Rice husk‐derived SiO_2_ NPs have been integrated with rare earth ions for in vivo bioimaging applications.^[^ ^123^ ^]^ Moreover, SiO_2_ NPs have been used as nanocarriers with chitosan and doxorubicin for tumor therapy and the efficacy has been evaluated in a mice model in vivo (Figure 3d).^[^ ^124^ ^]^

#### 2.2.2 Bioactive Ceramics from Agro‐Processed Residues

In addition to raw agricultural waste, agro‐processed waste can be recycled into SiO_2_‐based bioceramics. One example is the beer bagasse from beer manufacturers. The residues from beer production contain about 55% cellulose and hemicellulose, 34% lignin, and some inorganic matter. Beer bagasse carries essential elements, such as Si, P, calcium (Ca), and Mg with traces of sodium (Na), K, and Fe,^[^ ^125^ ^]^ which can endow implants with osteoinductive properties. The biochar of beer bagasse has been reported to contain polymorph SiO_2_ and Ca‐Mg‐PO_4_ phases.^[^ ^125^, ^126^ ^]^ The scaffolds made from the biochar of beer bagasse have been found to exhibit an in vitro osteogenic behavior that is comparable with commercial HA.^[^ ^126^ ^]^ In a 21 day subcutaneous in vivo study, the recycled biomaterial did not show any sign of foreign body rejection and allowed for tissue growth into the pores of the scaffold.^[^ ^127^ ^]^

Transforming agricultural residues into SiO_2_ bioactive ceramics offers a more cost‐effective and energy‐efficient alternative to synthetic SiO_2_ production. Notably, this approach significantly reduces the cost of raw material. In addition, the extraction of SiO_2_ from agricultural residues requires a lower temperature as compared to traditional synthetic production methods, typically around 1800 °C.^[^ ^128^ ^]^ The organic components present in these residues contribute to the formation of a natural porous microstructure within the SiO_2_.^[^ ^37^ ^]^ While rice husk, a readily available source of SiO_2_, is predominantly available in rice‐growing countries, other agricultural residues that contain varying percentages of SiO_2_ can be utilized by other countries. For example, wheat straw contains 50–55% SiO_2_, bamboo leaf contains 60–80% SiO_2_, sugarcane bagasse contains 50–97% SiO_2_, palm kernel shell contains 43% SiO_2_, groundnut shell contains 41% SiO_2_, and olive stone contains 32–46% SiO_2_.^[^ ^109^ ^]^ Furthermore, the use of agro‐processed residues promotes a more circular economy, enabling food and beverages companies to engage in a closed‐loop system and reduce their environmental footprint.

#### 2.2.3 Bioactive Compounds from Fruit and Vegetable Wastes

Fruits and vegetables are among the most widely consumed food products, accounting for 42% of the total food waste generated globally.^[^ ^129^ ^]^ Depending on their type, they exist in various forms, such as peels, seeds, crop, leaf, straw, stem, root, or tubers, throughout the stages of harvesting, production, and consumption. These commodities present significant opportunities for recycling of their corresponding wastes into a wide range of materials. Fruit and vegetable wastes can be transformed into antioxidants, vitamins, and fibers to improve a healthy human diet.^[^ ^130^ ^]^ They can also be used to produce biodegradable polymers for pharmaceutical applications and bioplastic films, which contribute to reducing the reliance on petroleum‐based packaging materials.^[^ ^131^ ^]^ Additionally, fruit and vegetable wastes can aid in the synthesis of nanoparticles for various biomedical applications.^[^ ^132^, ^133^ ^]^

### 2.3 Renewable and Recycled Biopolymers from Biomass

Natural polymers can be obtained from renewable resources or be recycled from waste.^[^ ^10^ ^]^ Natural polymers are often utilized in soft tissue repair (e.g., skin, muscle, and nerve tissue) or are loaded with bioactive compounds for the regeneration of various types of tissue, including bone.^[^ ^26^, ^27^, ^28^, ^29^, ^30^, ^31^, ^32^ ^]^ The surface morphology and geometries of the polymer scaffolds, their biodegradability, and the release profile of the bioactive agents can be modified by choosing different polymer chemistries and controlling the degree of cross‐linking. Natural polymers can be grouped into polysaccharide‐based (e.g., cellulose, chitosan, and alginate^[^ ^26^, ^27^, ^28^ ^]^) and protein‐based (e.g., collagen, silk, and keratin^[^ ^30^, ^31^, ^32^ ^]^).

#### 2.3.1 Cellulose Biomass

**Figure 4:**
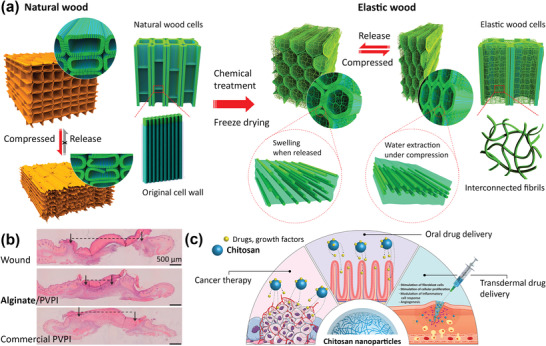
Functional renewable polysaccharide‐based natural polymers. a) Chemically treated, freeze‐dried wood recovers to its pristine state even when compressed to a high degree of strain. Adapted with permission.^[^ ^139^ ^]^ Copyright 2020, American Chemical Society. b) The H&E staining on untreated mice skin wound and those treated with alginate poly(vinylpyrrolidone)‐iodine (PVPI) and commercial PVPI, showing a faster in vivo wound closure in the biomaterial containing alginate. Adapted with permission.^[^ ^142^ ^]^ Copyright 2017, Elsevier. c) A schematic illustration of chitosan NPs for cancer therapy and as nanocarriers for oral and transdermal drug delivery. Reproduced with permission.^[^ ^146^ ^]^ Copyright 2022, Elsevier.

Cellulose is the most abundant natural polymer, found in plants and synthesized by bacteria. The global production of cellulose is estimated to be around 100 billion tons.^[^ ^134^ ^]^ Despite its large quantity, cellulose biomass is often undervalued although it has the potential to be an alternative biodegradable polymer or be used in composites that could replace fossil‐derived polymers. Research on the recycling of cellulose biomass has been performed,^[^ ^135^, ^136^ ^]^ as well as the use of nanocrystalline cellulose as the building blocks for materials, as cellulose nanofibers have high strength comparable to the strengths of steels and Kevlar, and high Young's modulus (138 GPa).^[^ ^137^, ^138^ ^]^ Cellulose and its derivatives have also been made into shape memory hydrogels (**Figure** **4**a) for various applications in soft robotics, energy storage, and tissue engineering.^[^ ^138^, ^139^ ^]^

#### 2.3.2 Renewable Alginate

Alginate is a biopolymer from brown algae. In 2019, about 30 000 tons of alginate were produced from 34.5 million tons of seaweeds.^[^ ^140^ ^]^ Alginate is an attractive marine‐based biopolymer that has the ability to form a gel in the presence of divalent ions, which makes it suitable for tissue engineering and pharmaceutical applications.^[^ ^27^, ^141^ ^]^ Alginate has also been used in combination with antiseptic compounds^[^ ^142^ ^]^ or curcumin extract^[^ ^143^ ^]^ for wound healing and skin tissue regeneration (Figure 4b). Moreover, alginate has been used in composites, e.g., in combination with collagen,^[^ ^144^ ^]^ for cartilage tissue repair.

#### 2.3.3 Chitosan from Crustaceans Shells and Fungi

Chitin is present in large amounts in the exoskeleton of crustaceans, making it a potential source of recycled material from the seafood industry.^[^ ^28^ ^]^ After deproteinization and demineralization of the exoskeletons, chitin can be isolated and deacetylated to form chitosan with various functionalities based on its molecular weight and degree of deacetylation. Chitin can also be produced through the fermentation of fungal waste obtained from the biotech industry.^[^ ^145^ ^]^ Fungal chitosan has been reported to be more homogenous, has a higher polydispersity and degree of deacetylation, and a lower molecular weight than chitosan from crustacean. Chitosan can be functionalized for drug delivery applications, including those for targeted cancer therapy (Figure 4c).^[^ ^146^ ^]^ Chitosan in combination with calcium phosphate has been studied for bone tissue engineering.^[^ ^147^ ^]^ Moreover, chitosan is inherently antimicrobial and has shown antibiofilm activity against methicillin‐resistant *Staphylococcus aureus* (MRSA),^[^ ^148^, ^149^ ^]^ making it a potential candidate for preventing implant‐associated infections.

#### 2.3.4 Collagen from Byproducts of Fishing Industries

**Figure 5:**
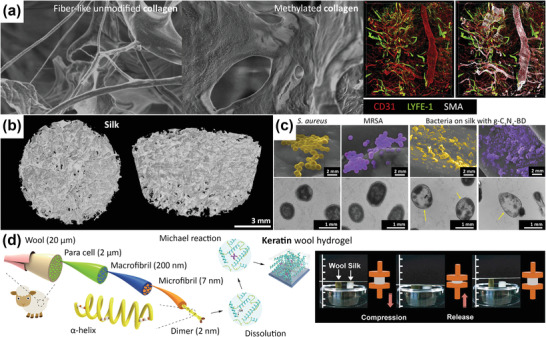
Functional renewable protein‐based natural polymers. a) The morphologies of fiber‐like unmodified fish scale‐derived collagen and methylated collagen. The methylated collagen patch, crosslinked with 1,4‐butanediol diglycidyl ether, enabled blood and lymphatic vessel regeneration recognized by the CD31 and LYVE‐1 staining around the smooth muscle actin which may be suitable for inflammation‐related disease treatment. Adapted with permission.^[^ ^160^ ^]^ Copyright 2017, Elsevier. b) 3D micro‐CT images of salt‐leached and freeze‐dried porous silk fibroin scaffolds. Reproduced with permission.^[^ ^165^ ^]^ Copyright 2011, Elsevier. c) Field‐emission scanning electron microscope (FE‐SEM) and transmission electron microscope images of *S. aureus* and MRSA in the membrane of the control group and flat silk cocoon with charged graphitic carbon nitride (g‐C_3_N_4_) and benzophenone tetracarboxylic dianhydride (BD). Reproduced with permission.^[^ ^167^ ^]^ Copyright 2022, Wiley‐VCH GmbH. d) A schematic illustration of highly elastic keratin hydrogel made of wool for flexible strain sensor applications. Adapted with permission.^[^ ^172^ ^]^ Copyright 2020, Wiley‐VCH GmbH.

Collagen makes up ≈30% of the total protein content in the human body. Collagen type I is most commonly found in human connective tissues, such as skin, bone, tendon, and ligament.^[^ ^150^ ^]^ Its biomimetic properties makes it an attractive component for the extracellular matrix in tissue engineering.^[^ ^150^, ^151^ ^]^ Traditionally, collagen has been often obtained from livestock byproducts like bovine tendon and porcine skin. However, the use of livestock‐derived collagen has become limited due to the risk of disease transmission.^[^ ^152^ ^]^ An alternative, sustainable source of collagen is the byproducts of the fishery and fish‐processing industries, including fish skin, jellyfish, sea urchins, starfish, and sponges.^[^ ^153^, ^154^ ^]^ To isolate collagen from the fish by‐products, first, alkali (e.g., using NaOH) and alcohol (e.g., butyl alcohol or ethanol) pretreatments are performed to remove noncollagenous proteins, fats, and pigments. For skeletal byproducts, a demineralization process is required, e.g., using ethylenediaminetetraacetic acid. Then, the collagen can be extracted using acid or enzyme, such as pepsin.^[^ ^30^, ^155^ ^]^ Scaffolds and composites made of collagen derived from marine resources have been demonstrated to have the potential to stimulate tissue regeneration in vivo, such as bone,^[^ ^156^, ^157^, ^158^ ^]^ skin,^[^ ^159^ ^]^ and blood and lymphatic vessels (**Figure** **5**a).^[^ ^160^ ^]^

#### 2.3.5 Renewable Silk Fibroin

Silk is a renewable fiber material that is produced by silkworms. Once the silkworms metamorphose and emerge from the cocoon, the remaining cocoons can be collected and processed into sustainable silk fibers.^[^ ^161^ ^]^ Although the traditional method of extracting silk fibroin (or degumming processes using N_a2_CO_3_ alkali treatment) requires significant amounts of water and energy, several environmentally friendly degumming processes have been developed which use alternative approaches, such as enzymes, CO_2_ supercritical fluid, steam, or ultrasonic processes.^[^ ^162^ ^]^ Biomaterials made of silk fibroin (Figure 5b) can aid in the repair of various types of tissues, such as bone^[^ ^31^, ^163^, ^164^ ^]^ and articular cartilage,^[^ ^165^ ^]^ including infection prevention and treatment (Figure 5c).^[^ ^166^, ^167^, ^168^ ^]^

#### 2.3.6 Keratin from Feathers and Wools

Keratin is a protein that is found in the outer layers of skin, hair, and nails. It has a high mechanical strength due to the peptide bonds, making it suitable for various applications, such as adhesives, fibers, and films.^[^ ^169^ ^]^ The poultry industry generates a large amount of waste in the form of chicken feather, while the coarse wool fibers are considered waste in textile industry.^[^ ^32^ ^]^ These biomass can be used as a source of keratin, which can be extracted using green methods, such as enzymatic or chemical‐free hydrolysis.^[^ ^170^ ^]^ Keratin from chicken feathers,^[^ ^171^ ^]^ wool,^[^ ^172^, ^173^, ^174^ ^]^ and human hair^[^ ^175^, ^176^ ^]^ has been developed into hydrogels for wound dressing, bone scaffolds, as well as wearable and implantable medical devices (Figure 5d).

As far as biomedical applications are concerned, renewable and recycled biopolymers offer a distinct advantage, namely, improved biocompatibility, over synthetic polymers.^[^ ^177^ ^]^ Cellulose, chitosan, alginate, silk fibroin, and keratin have been widely applied for tissue engineering applications.^[^ ^178^, ^179^, ^180^, ^181^ ^]^ However, one of their main drawbacks is the lack of sites for cell adhesion. These polymers, unlike collagen, require a conjugation of specific proteins, such as Arg‐Gly‐Asp (RGD) peptides, to provide cell binding sites.^[^ ^179^, ^180^ ^]^ Furthermore, it is important to note that biopolymers alone may not be sufficient for bone tissue repair due to the absence of bony minerals. A combination with other materials (e.g., injectable alginate hydrogels containing akermanite and glutamic acid^[^ ^182^ ^]^ or Bio‐Oss‐collagen scaffolds^[^ ^183^ ^]^) can be useful to better mimic the mechanical properties of the native bone and promote bone regeneration.

### 2.4 Probiotics

**Figure 6:**
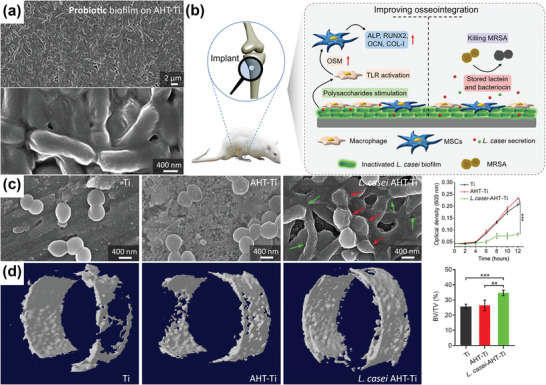
Probiotics for bone tissue regeneration and infection prevention. a) inactivated *L. casei* biofilm coating on alkali heat‐treated (AHT)‐Ti implants. b) A schematic illustration of using inactivated *L. casei* biofilm for MRSA infection prevention and simultaneously improving osseointegration. c) SEM images of the morphologies of MRSA after being cultured on Ti, AHT‐Ti, and *L. casei* biofilm AHT‐Ti specimens and the quantitative growth of MRSA over time. d) The micro‐CT reconstruction of new bone tissue growing around the implants with quantitative new bone volume fractions. Adapted under the terms of the CC‐BY‐NC 4.0 license.^[^ ^191^ ^]^ Copyright 2020, The Authors. Published by American Association for the Advancement of Science.

Beneficial microbes, known as probiotics, have been shown to influence human health, including bone biology, through several mechanisms.^[^ ^184^, ^185^ ^]^ A diet rich in probiotics modulates the immune response, leading to changes in bone mass density.^[^ ^186^ ^]^ Probiotics can increase the production of short chain fatty acids,^[^ ^187^ ^]^ which can improve mineral solubility and balance systemic Ca levels.^[^ ^188^ ^]^ Some probiotic strains also synthesize vitamins, such as vitamin K2, which plays a role in bone mineralization.^[^ ^189^ ^]^ Probiotics can be sustainably cultured using agricultural waste or food waste from households or industries. However, careful selection of waste materials based on the nutrient content, compatibility with probiotic strains, and ensuring proper hygiene during fermentation are all essential. Even though probiotics have a potential as renewable materials benefiting bone tissue regeneration, directly administering living probiotics to bone injury sites may entail the risks of bacteremia and sepsis.^[^ ^190^ ^]^ Probiotics, such as *Lactobacillus casei*, have been cultured on the surface of Ti implant to form biofilms, and then inactivated using UV irradiation (**Figure** **6**a).^[^ ^191^ ^]^ The biofilm of inactive *L. casei* stimulated macrophages to produce osteogenic factors accelerating osseointegration, while simultaneously preventing MRSA infection (Figure 6b–d).^[^ ^191^ ^]^

### 2.5 Recycled Metals

Metal recycling is a well‐established industry that plays a significant role in the global economy. It is an efficient and environmentally friendly way of preserving natural resources, minimizing energy consumption, and reducing greenhouse gas emissions. Nevertheless, limitations in the recycling technologies or the thermodynamic separation of materials can make the process inefficient or even impossible.^[^ ^192^ ^]^ Currently, ferrous metals, such as stainless steel, have a recycling rate of over 60%, while other non‐ferrous metals, such as aluminum (Al), Cu, and lead (Pb), have recycling rates of >33%, >40%, and >35%, respectively.^[^ ^193^ ^]^

Unlike the aforementioned metals, Ti has a low recycling rate of <1%,^[^ ^194^ ^]^ despite its widespread use in the aerospace, automotive, medical (i.e., as bone implants), and energy industries. Collecting a sufficient volume of end‐of‐life Ti products for recycling is challenging, due to its long lifespan. Other metals, such as Mg, have been recycled too, despite the susceptibility of Mg to oxidation, which makes its recycling highly challenging.^[^ ^195^ ^]^ The presence of impurities, such as Fe, Cu, nickel (Ni), and Si, can hinder the recycling process. Maintaining the purity of Mg and its rare alloying elements is another challenge to be addressed.^[^ ^195^ ^]^ As a result, Mg is typically downcycled into secondary products for use in the production of Al alloys or cast Fe. Similar to Mg, Zn has been primarily downcycled into secondary products to support its increasing global demand.^[^ ^196^ ^]^

The development of technologies in metal recycling aims to reduce the dependency on Earth's natural resources. However, it is not yet at a stage where it can replace all primary material resources, especially for manufacturing medical devices, which require a high level of purity control.

## 3 Additive Manufacturing of Sustainable Bone Implants

### 3.1 Additive Manufacturing of Recycled Calcium Phosphate Bioceramics

Studies on AM of eggshell, seashell, and fish bone powders are limited. Most studies embedded the recycled powder into a polymer matrix as composite filaments for AM purposes. Eggshell powder (<25 µm), seashell powder (<50 µm), and fish bone powder (<75 µm) have been mixed with polylactic acid (PLA) granules.^[^ ^197^, ^198^, ^199^ ^]^ The addition of 4 wt% eggshell and 10 wt% seashell powder has been shown to improve the tensile and compressive mechanical properties of PLA.^[^ ^197^, ^198^ ^]^ Meanwhile, the addition of 10 wt% fish bone powder reduced the tensile strength of the PLA composite filaments due to the release of oil from the raw fish bone which acted as a plasticizer.^[^ ^199^ ^]^

Oyster shell powder (<63 µm) and fish bone‐derived HA powder have been added to strengthen PCL scaffolds. The addition of 10 wt% oyster shell powder to PCL has been found to improve the compressive strength and in vitro proliferation and alkaline phosphatase (ALP) activity of MG‐63 cells.^[^ ^200^ ^]^ Moreover, PCL containing 10 wt% fish bone‐derived HA, which was also coated with 2% fish collagen, improved the in vivo bone formation in mouse calvaria defect as compared to PCL in its pristine state.^[^ ^201^ ^]^ In another study, PCL scaffolds were coated with 3% fish bone extracts and exhibited better cell proliferation and resulted in an enhanced expression of osteogenic markers (i.e., bone morphogenetic protein (BMP)−2, ALP, osteopontin (OPN), and osteocalcin (OCN)) by MC3T3‐E1 cells.^[^ ^202^ ^]^

To date, only two studies have reported AM using raw eggshell powder and seashell powder precursors. In one such study, eggshell powder (<100 µm) was dispersed in H_3_PO_4_ (at a mass ratio of 1:5) and ball‐milled before mixing with 6% chitosan binder (at a mass ratio of 2:1).^[^ ^203^ ^]^ The eggshell inks were 3D printed into porous scaffolds, heat treated at 400 and 600 °C for 2 h each and at 800 °C for 1 h.^[^ ^203^ ^]^ In another study, seashell inks were prepared from seashell powder (<100 µm) with H_3_PO_4_ (in a mass ratio of 1:5) and 4% chitosan binder. The seashell inks were extruded into 3D porous scaffolds, followed by a sequential heat treatment at 400, 600, and 800 °C for 2 h each and at 900 °C for 1 h.^[^ ^204^ ^]^

**Figure 7:**
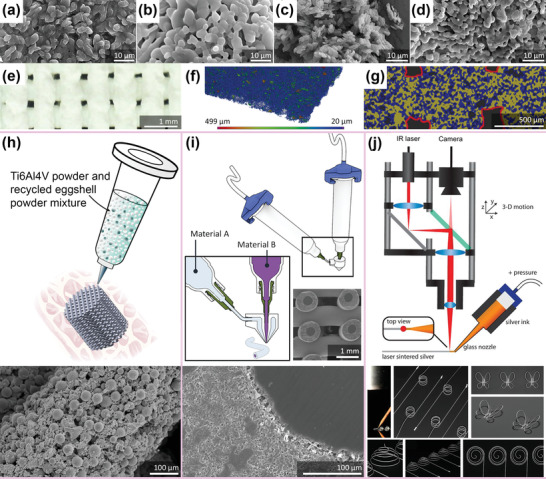
AM of biomaterials using recycled materials. a,b) The surface morphologies of AM eggshell‐derived calcium phosphate. Reproduced with permission.^[^ ^203^ ^]^ Copyright 2016, American Chemical Society. c,d) The surface morphologies of AM seashell‐derived calcium phosphate. Reproduced with permission.^[^ ^204^ ^]^ Copyright 2021, American Chemical Society. e) The top view of 3D‐printed monolithic SiO_2_ made from rice husk ash precursor, f) a 3D model of the scaffold, and g) the µ‐CT images showing the macropores of SiO_2_ scaffolds and the interconnected micropores in the struts. Adapted with permission.^[^ ^206^ ^]^ Copyright 2022, Elsevier. h) A schematic illustration of extrusion‐based AM using an ink containing multiple materials (e.g., Ti6Al4V and eggshell powder) and an SEM image of Ti6Al4V‐CaO composite scaffolds (original research reported for the first time here). i) A schematic illustration of coaxial 3D printing of multimaterial (Adapted with permission. ^[^ ^237^ ^]^ Copyright 2021, Wiley‐VCH GmbH) with SEM images of TCP (shell) and polycaprolactone (core) composite scaffold (Adapted with permission.^[^ ^236^ ^]^ Copyright 2019, Elsevier). j) A schematic illustration of laser‐assisted direct ink writing of silver ink and examples of the 3D‐printed structures. Reproduced with permission.^[^ ^238^ ^]^ Copyright 2016, National Academy of Sciences.

Both AM eggshell and AM seashell calcium phosphate scaffolds exhibited a granular surface microstructure (**Figure** **7**a–d) and in vitro cytocompatibility with mesenchymal stem cells. They also improved osteogenic differentiation as compared to TCP.^[^ ^203^, ^204^ ^]^ In subcutaneous in vivo tests, the eggshell‐derived calcium phosphate exhibited ectopic bone formation,^[^ ^203^ ^]^ while the seashell‐derived calcium phosphate initiated endochondral ossification.^[^ ^204^ ^]^ Despite their promising biological performance, both scaffolds were characteristically brittle, which limits their use in load‐bearing applications.^[^ ^203^, ^204^ ^]^

### 3.2 Additive Manufacturing of Recycled Silica and Bioactive Bioceramics

Research on AM of agricultural waste precursors (i.e., rice husk), potentially for tissue engineering, has only recently appeared in the literature.^[^ ^205^, ^206^ ^]^ Extrusion‐based AM was employed to fabricate the architected monolithic SiO_2_ foam (Figure 7e) with a high total porosity (i.e., ≈89%) and a tailorable pore structure (Figure 7f,g).^[^ ^206^ ^]^ This lightweight rice husk‐derived SiO_2_ foam has the potential to be studied as a controlled on‐demand drug delivery system for tissue regeneration. Another study made use of the same AM technique to fabricate porous mullite scaffolds, made of rice husk ash‐derived SiO_2_ powder and alumina (Al_2_O_3_) powder. These scaffolds have gained attention for industrial applications as high‐temperature structural components, acoustic materials, and implantable medical devices.^[^ ^205^ ^]^ Other studies have prepared composite filaments made of PLA^[^ ^207^, ^208^ ^]^ and polypropylene,^[^ ^209^ ^]^ containing rice husk biochar powder for AM purposes.

While rice husk biochar contains a large amount of SiO_2_, the biochar of other agricultural residues mainly contains carbon black with additional minerals, such as K, Mg, Zn, Fe, Cu, and manganese (Mn).^[^ ^210^ ^]^ Various AM technologies have been used to fabricate carbon‐based materials with different types of feedstock materials, such as pure carbon, carbon black, carbon fiber, carbon nanotube, graphene, and their composites.^[^ ^211^ ^]^ The AM of carbon‐based materials has been reviewed elsewhere for their unprecedented applications in the energy sector and tissue engineering, including the use of biomass as the sustainable resource.^[^ ^212^, ^213^, ^214^ ^]^

### 3.3 Additive Manufacturing of Renewable and Recycled Biopolymers

AM of natural polymers (e.g., cellulose,^[^ ^215^ ^]^ alginate,^[^ ^216^ ^]^ chitosan^[^ ^217^ ^]^) and proteins (e.g., collagen,^[^ ^218^ ^]^ silk,^[^ ^219^ ^]^ and keratin^[^ ^220^ ^]^) has been widely studied and reviewed, particularly in the context of 3D bioprinting of tissue and organs. Natural polymers and protein‐based bioinks, as well as their combinations, have been developed and have been shown to influence multiple cellular and molecular interactions, histogenesis, and tissue and organ maturation. These bioinks provide chemical, mechanical, and physical properties that mimic specific tissues. They are also biodegradable and promote the host‐material integration, which is promising for application in tissue engineering and regenerative medicine. The ink formulation, 3D printability of bioinks, and assessment of the shape fidelity and functions of the scaffolds have been reviewed in detail elsewhere.^[^ ^221^, ^222^, ^223^ ^]^ The development of bioink formulation must take such factors into consideration as the possible interactions between the biopolymer and precrosslinking compounds in the feedstock before, during, and after printing. The viscosity and rheological behavior, as affected by composition and temperature, should be optimized to ensure flowability, while preventing excessive shear to ensure the survival of cells in the bioinks. The choice of biopolymers should match the preliminary and secondary crosslinking strategies so as to obtain better‐defined scaffold shapes and improved functionalities for various tissue engineering applications. Furthermore, the combination of these sustainably sourced polymers and proteins bioinks with recycled inorganic materials can further improve the biofunctionality of the materials.

### 3.4 Additive Manufacturing of Recycled Metals

AM of metals using recycled metal powders has not been demonstrated yet. The current focus of metal recycling lies on recycled bulk materials for various manufacturing industries. There are still several challenges that need to be addressed before shifting the focus toward recycling metals for the generation of powder feedstock to be used in AM.

While the standardized commercial medical‐grade Ti6Al4V alloy powder is available, there are no such standardized commercially available Fe, Mg, and Zn powders for AM of biodegradable implants.^[^ ^224^ ^]^ There is, therefore, a need to develop and standardize such metallic powders, preferably starting from metal waste streams to enable the streamlined production of AM biodegradable implants from recycled metals.

## 4 Challenges in Recycling Waste Materials

Recycling waste and residual materials for added value is an essential process that helps in reducing waste and minimizes the use of primary resources obtained from the Earth. However, a number of challenges need to be addressed to ensure that the recycling process runs efficiently. The first challenge is related to the proper collection and sorting of waste materials. Eggshells and feathers can be obtained from poultry, while agricultural waste can be gathered from local farmers. The residues of crustacean shells, fish, seashells, and seaweed can be obtained from the fishing industry. To ensure the efficiency, the waste and residues should be collected directly before they are sent to landfills or incineration.

The mineral composition, color, and thickness of eggshells can vary depending on the breed, feed, and overall well‐being of hens.^[^ ^33^ ^]^ To minimize such variations, eggshell waste should be gathered from the same stakeholders, e.g., a group of poultry farmers who care for their hens under similar conditions. Different species of crustacean shell, fish bone, fish scale, and seashell also have varying compositions of minerals, polysaccharides, and protein content.^[^ ^225^ ^]^ The trace elements present in different species of fish bone have been found to influence the final phases of synthesized calcium phosphate bioceramics.^[^ ^226^ ^]^ Therefore, the waste of crustacean shells, fish residues, seashells, and seaweeds should be grouped based on the type or species. Additionally, to ensure a consistent molecular weight and chemical composition of natural polymers and materials derived from agricultural residue, it is important to use a similar source of biomass for the processing step.

The next challenge is to ensure that the waste materials are thoroughly cleaned to remove any organic matter or contaminants. Eggshells may contain residual egg white or yolk, while seashells or seaweeds may carry sea debris, and agricultural residues may contain dirt. Cleaning fish residues is particularly demanding, as the fish parts must be separated (e.g., scale, head, tail, and internal organs). Then, the fish scale and fish bone must be cleaned from the remaining flesh to prevent odor and microbial contaminations. Once the waste materials are cleaned, they should be sterilized to eliminate pathogenic contaminations.

After the cleaning step, each type of waste material should undergo standardized processing to achieve a consistent chemical composition, particle sizes, and particle shape. Agricultural residues can be pyrolyzed to obtain the biochar which contains valuable inorganic compounds, as well as biodiesel and syngas. Depending on the type of the pyrolysis process and its parameters, the ratios of biochar, biofuel, and biogas can be fine‐tuned to enhance the value of recycled agricultural residues.^[^ ^227^, ^228^ ^]^ To maximize the utilization of fish residues, such as fish bone and fish scale, first, collagen should be extracted. The remaining inorganic components can then be refined into calcium phosphate materials.^[^ ^229^ ^]^ Finally, the biochar from agricultural residues, the inorganic components of fish residues, eggshells, and seashells, can be ground and sieved into powder with a desired range of particle sizes for further processing.

As for recycling polymers from its biomass and processing the renewable polymers, typically chemical and enzymatic processes are involved. The pH, temperature, catalyst concentration, and reaction time of the process can be optimized to obtain polymers with specific physical and chemical properties.^[^ ^230^ ^]^ Then, the polymer is filtered and dried into the powder form, ready for use. After the waste and residual materials have been collected, sorted, cleaned, and processed, it is important to conduct regular quality control. This will ensure that the recycled materials meet the standards and requirements, particularly for use in medical devices.

To translate the use of waste‐derived biomaterials into clinical applications, these biomaterials must comply with the provisions of the medical devices regulation in Europe or receive approval from the US Food and Drug Administration. The classification of the waste‐derived biomaterials should be accurately determined based on their characteristics, intended medical application, and risk profile. Additionally, the biomaterials must undergo a conformity assessment. It is necessary to prepare a comprehensive set of technical documentation that covers the entire process chain, starting from the initial waste recycling steps to biomaterial design and manufacturing, in vitro and in vivo evaluations, clinical tests, and safety assessments. The waste collection, sorting, and classification processes pose the most significant challenges for these biomaterials. This critical stage determines the purity of the resulting material for subsequent use in medicine. Once the recycling step is completed effectively, waste‐derived biomaterials can undergo the approval process similar to other synthetic biomaterials currently utilized in medicine.

A wide range of medical and pharmaceutical grade biopolymers and bioceramics is currently available in the market. One such example is SeriTech Company Ltd., which produces pharmaceutical‐grade silk fibroin proteins. Medical‐grade chitosan and collagen have been extensively developed for wound dressing applications, exemplified by such products as ChitoGauze XR Pro and Integra Wound Matrix. Other companies, such as Geistlich develops Bio‐Oss bone substitutes while ZimVie develops RegenerOss bone graft plug made of collagen and IngeniOs HA or TCP bioceramics particles.

## 5 Discussion and Future Perspective

The current state‐of‐the‐art in recycling waste materials into biomaterials, including bone substitutes, is continuously evolving. Various approaches, such as ball milling and chemical pretreatment, hydrothermal treatment, pyrolysis, and enzymatic hydrolysis, have been developed with the aim of improving the efficiency and yield in repurposing waste materials. While these methods have shown promising results, AM technologies have only recently been introduced in the production of biomaterials from sustainable sources of raw materials.

Among all AM technologies, direct ink writing (DIW)—an extrusion‐based 3D printing technique, has been used for AM of biomaterials using sustainable resources, such as eggshells, seashells, and rice husk biochar.^[^ ^203^, ^204^, ^205^, ^206^ ^]^ This particular AM technique has been known for its versatility in multiple material fabrication,^[^ ^231^ ^]^ e.g., for permanent implants made of Ti6Al4V‐β‐TCP^[^ ^232^ ^]^ and biodegradable implants made of MgZn‐β‐TCP^[^ ^233^ ^]^ and FeMn‐akermanite.^[^ ^234^ ^]^ Although extrusion‐based 3D printing of calcium phosphate made from eggshells and seashells and rice husk‐derived SiO_2_ foams has been successful, further studies and improvements are required to achieve properties that are required for their application as bone implants particularly when the implants are to be used as load‐bearing devices.

The present challenges in AM of biomaterials using sustainable resources can be addressed by leveraging the multimaterial capability of extrusion‐based 3D printing. We propose several possibilities using the currently available extrusion‐based 3D printing technique.

- A 3D printable ink containing multiple powder materials can be prepared, extruded into scaffolds (Figure 7h), and post‐processed with optimized debinding and sintering parameters. For example, eggshell powder can be mixed with Ti6Al4V or other metallic powders and be 3D printed using extrusion‐based techniques into metal–ceramic composite implants (Figure 7h). Such implants possess bioactive properties offered by the eggshell‐derived CaO, while the load‐bearing performance is guaranteed by the use of sufficient percentage of a porous metal matrix.
- Two separate 3D printable inks made of metal powder (e.g., Ti6Al4V) and ceramic powder (e.g., eggshell powder) can be prepared, extruded co‐axially, and post‐processed to obtain scaffolds with bi‐material strut‐based microarchitectures. The resulting scaffolds will have eggshell‐derived CaO in the core of the strut and Ti6Al4V as the shell. This configuration can be benefitted from the strength and ductility of the metallic shell, while the brittle CaO ceramic in the core of the struts may provide strength enhancement. The Ti6Al4V in the shell of the strut can be tuned to have a certain degree of porosity, allowing the CaO core to biodegrade, thereby leaving additional space for bone ingrowth. Although scaffolds made from core–shell metal‐ceramic strut configurations have not yet appeared in the literature, scaffolds made from core–shell calcium magnesium silicate‐β‐TCP^[^ ^235^ ^]^ and β‐TCP‐polycaprolactone^[^ ^236^ ^]^ (Figure 7i) have been reported. Currently, the coaxial nozzle‐based AM technique is mostly utilized for the fabrication of vascular networks, microfluidic applications, or flexible, stretchable, conductive, and piezoelectric sensors.^[^ ^237^ ^]^
- In situ DIW involving laser for selectively melting or sintering during the extrusion of ink material can be utilized. Laser‐assisted DIW has been reported for the fabrication of 3D structures made of a silver ink (Figure 7j).^[^ ^238^ ^]^ However, this has not yet been utilized for multimaterial fabrication of scaffolds intended for bone tissue engineering or with recycled materials as the feedstock. This concept could further advance the AM of multiple materials, including the possibility of delivering hydrogel coatings made of natural polymers right after the in situ AM of multimaterial metal–ceramic composites that are currently not yet available.

The progress made in repurposing waste materials for sustainable biomaterials and medical devices is promising, but further development is needed. This includes streamlining the waste recycling process with state‐of‐the‐art AM technologies, optimizing the properties of waste‐derived biomaterials, and further assessing their biocompatibility.

Typically, the recycling process involves converting waste materials into powder, pellet, paste, or liquid form, then chemically pretreating them into desired biomaterial phases, followed by a fabrication step where the raw materials are converted into medical devices. These three steps can be reduced to two by using extrusion‐based AM technologies. The binder composition in the ink can accommodate relevant chemicals that convert the initial waste powder materials into the desired phase compositions during 3D printing, thereby incorporating the chemical pretreatment step into the fabrication step. For example, raw CaCO_3_ powder from eggshells or seashells can be mixed with a binder comprising of tetraethyl orthosilicate, followed by extruding and sintering into CaSiO_3_ bioceramic implants in a single step.

Optimizing the properties of materials developed from sustainable raw materials should follow the established blueprints for various clinically approved biomaterials and medical devices. By building on the existing work, the process can be more efficient. For example, the development of Ca‐rich waste shares similarities to the research on HA or TCP. In addition to chemistry, the physical structures of medical devices can follow the design library available for AM structures for various applications, including complex porous geometry of bone substitutes.

While various waste materials, such as eggshells, bioactive char, crustacean shells, fish residues, and seashells have been shown to be biocompatible, more detailed in vitro and in vivo studies are required to ensure their safety for clinical application. In the case of bone implants, it is important to check the inflammatory responses of the materials as well as their potential to be antimicrobial (e.g., a bacteriostatic substrate), in addition to the osteogenic properties of such materials.

Finally, recycling and repurposing waste into functional biomaterials and medical devices not only improve the value and life cycle of the materials but also reduce the energy consumption and waste generated by hospitals. Future research should maximize the use of waste materials that provide avenues for repurposing biomaterials and medical devices to ensure sustainability.

## 6 Conclusions

Renewable materials, as well as various types of waste materials, can be recycled to reduce the impact of industrial activities on the environment and reduce our dependency on primary material resources. Transforming waste materials, such as eggshells, seashells, residues from crustacean and fish, agricultural residue, and polysaccharide and protein biomass, into biomaterials for bone tissue engineering is a well‐established practice. Meanwhile, the use of recycled metals in the fabrication of biomaterials is in its infancy. The AM of biomaterials from renewable and recycled resources has only been recently implemented. Although the preliminary results are promising, further research will be needed to advance the progress toward clinical applications.

## Conflict of Interest

The authors declare no conflict of interest.
